# *In Vivo* Manganese Exposure Modulates Erk, Akt and Darpp-32 in the Striatum of Developing Rats, and Impairs Their Motor Function

**DOI:** 10.1371/journal.pone.0033057

**Published:** 2012-03-13

**Authors:** Fabiano M. Cordova, Aderbal S. Aguiar, Tanara V. Peres, Mark W. Lopes, Filipe M. Gonçalves, Aline P. Remor, Samantha C. Lopes, Célso Pilati, Alexandra S. Latini, Rui D. S. Prediger, Keith M. Erikson, Michael Aschner, Rodrigo B. Leal

**Affiliations:** 1 Departamento de Bioquímica, Centro de Ciências Biológicas, Universidade Federal de Santa Catarina, Florianópolis, Brazil; 2 Departamento de Farmacologia, Universidade Federal de Santa Catarina, Florianópolis, Brazil; 3 Centro de Ciências Agroveterinárias, Universidade do Estado de Santa Catarina, Lages, Brazil; 4 Centro de Ciência Animal, Universidade Federal do Tocantins, Araguaína, Brazil; 5 Department of Nutrition, University of North Carolina, Greensboro, North Carolina, United States of America; 6 Department of Pediatrics, Vanderbilt University Medical Center, Nashville, Tennessee, United States of America; Massachusetts Eye & Ear Infirmary - Harvard Medical School, United States of America

## Abstract

Manganese (Mn) is an essential metal for development and metabolism. However, exposures to high Mn levels may be toxic, especially to the central nervous system (CNS). Neurotoxicity is commonly due to occupational or environmental exposures leading to Mn accumulation in the basal ganglia and a Parkinsonian-like disorder. Younger individuals are more susceptible to Mn toxicity. Moreover, early exposure may represent a risk factor for the development of neurodegenerative diseases later in life. The present study was undertaken to investigate the developmental neurotoxicity in an *in vivo* model of immature rats exposed to Mn (5, 10 and 20 mg/kg; i.p.) from postnatal day 8 (PN8) to PN12. Neurochemical analysis was carried out on PN14. We focused on striatal alterations in intracellular signaling pathways, oxidative stress and cell death. Moreover, motor alterations as a result of early Mn exposure (PN8-12) were evaluated later in life at 3-, 4- and 5-weeks-of-age. Mn altered in a dose-dependent manner the activity of key cell signaling elements. Specifically, Mn increased the phosphorylation of DARPP-32-Thr-34, ERK1/2 and AKT. Additionally, Mn increased reactive oxygen species (ROS) production and caspase activity, and altered mitochondrial respiratory chain complexes I and II activities. Mn (10 and 20 mg/kg) also impaired motor coordination in the 3^rd^, 4^th^ and 5^th^ week of life. Trolox™, an antioxidant, reversed several of the Mn altered parameters, including the increased ROS production and ERK1/2 phosphorylation. However, Trolox™ failed to reverse the Mn (20 mg/kg)-induced increase in AKT phosphorylation and motor deficits. Additionally, Mn (20 mg/kg) decreased the distance, speed and grooming frequency in an open field test; Trolox™ blocked only the decrease of grooming frequency. Taken together, these results establish that short-term exposure to Mn during a specific developmental window (PN8-12) induces metabolic and neurochemical alterations in the striatum that may modulate later-life behavioral changes. Furthermore, some of the molecular and behavioral events, which are perturbed by early Mn exposure are not directly related to the production of oxidative stress.

## Introduction

Manganese (Mn) participates in several biological processes, with important roles in regulating metabolism [1]. In the central nervous system (CNS), Mn is an important co-factor for several enzymes, including superoxide dismutase (SOD) and glutamine synthetase (GS) [2]. However, excessive exposure to Mn is neurotoxic, resulting in a neurodegenerative disease affecting cortical structures and basal ganglia, specifically the globus pallidus, striatum and substantia nigra pars reticulata [3], [4]. Cytotoxicity associated with excessive Mn exposure leads to neurological dysfunction associated with dystonic movements, analogous to those commonly noted in idiopathic Parkinson's disease (PD) [5]. Notably, much less is known about the effects of Mn on the developing CNS, in particular potential risks associated with early Mn exposure and predisposition to later-life onset neurological injury [6]–[9]. Newborns retain greater Mn levels than adults [7] and the developing brain is more susceptible to injury caused by toxic agents [10]–[12], reflecting immature and inefficient homeostasis, low physiological Fe levels and a permeable blood-brain barrier [5], [6], [13], [14]. The CNS continues to develop postnatally, and its vulnerability remains high for an extended period of time, from childhood to adolescence. Although many neurons are formed at birth, a substantial acceleration in RNA, DNA and protein synthesis, neuronal migration, glial cells growth (particularly astroglia, the main site for glutamate and metal uptake) and axonal myelination persist for several months into the postnatal period [10], [15], [16].

Mn exposure is commonly associated with occupational and industrial processes [4], [5], [7], [17]. High Mn concentrations are found in the environment due to its abundance in the earth's crust and secondary to its use in water treatment, manufacturing of dry batteries, as well as addition to gasoline (as an antiknock agent; methylcyclopentadienyl manganese tricarbonyl, MMT) and fungicides [3], [6], [18], [19]. Infantile Mn exposure is also associated with parenteral nutrition, which is commonly supplemented with excessive Mn levels [14], [20], [21].

Mn accumulation in the CNS is regulated by several transport mechanisms, including divalent metal transporter 1 (DMT-1) and transferrin/transferrin receptor system (Tf/TfR). However, it has yet to be ascertained which mechanisms regulate Mn transport during the developmental period [6]. Notably, both DMT-1 and TfR are expressed in rat cortex, hippocampus and striatum on the fifth postnatal day [13], [22]. Additionally, in the immature brain Mn may be transported by other mechanisms, such as ZIP-8 solute carrier, calcium channels, ionotropic glutamate receptors and the dopamine transporter, as previously shown in the adult brain [19], [23]–[27].

Mechanisms involved in Mn neurotoxicity are poorly understood [5]–[7], [28]. Oxidative stress is considered to play a major role, secondary to enhanced levels of redox-active Mn ions [29]. Mn toxicity is mediated by mitochondrial perturbations, initiating both apoptotic and necrotic cell death through the formation of reactive oxygen species (ROS) and oxidative stress [30]–[34]. Additionally, Mn induces dopamine autoxidation, increasing the levels of toxic quinines [35]. Higher sensitivity of the striatum to Mn-induced oxidative stress [36], especially during development, has been noted [6]. Oxidative stress in the striatum is associated with impairment of motor activity [37]. It has also been shown that Mn-dependent increased ROS formation can interfere with the removal of glutamate from the synaptic cleft [6], [38], resulting in excitotoxicity [6], [39].

In addition, many signaling pathways associated with programmed cell death are activated after *in vitro* Mn treatments, including JNKs, ERK1/2, p38^MAPK^, PKC and caspases [40]–[52]. However, the modulation of signaling pathways by Mn has yet to be shown in a systematic manner in the *in vivo* developing CNS.

The protein kinases ERK1/2, JNK1/2/3 and p38^MAPK^ are the foremost enzymes studied in the MAPK family [53]–[56]. The ERK1/2 cascade is primarily activated by growth factors, regulating gene expression, embryogenesis, proliferation, cell death/survival and neuroplasticity [54], [55]. The JNK1/2/3 and p38^MAPK^ protein kinases, classically recognized as stress-activated protein kinases (SAPKs), are activated by cytokines and cytotoxic insults, and are often related to stress and cell death [57], [58]. However, JNK and p38^MAPK^ also regulate CNS development and neuroplasticity [56], [59]. Another important intracellular signaling pathway is PI3K/AKT (PKB) which can be activated by several growth factors. It plays a central role in cell growth regulation, proliferation, metabolism and cell survival, as well as neuroplasticity [60], [61].

The basal ganglia receive inputs mainly through the striatum and coordinate vital behaviors, including movement, reward and motivational processes [62]. Corticolimbic-thalamic glutamatergic and mesencephalic dopaminergic systems converge on the gamma aminobutyric acid (GABAergic) medium-sized spiny neurons of the striatum [63]. These neurons characteristically express a dopamine- and cAMP-regulated phosphoprotein of 32 kDa (DARPP-32) [64], [65]. DARPP-32 function depends on its relative state of phosphorylation in two main regulatory sites, Thr-34 and Thr-75. When DARPP-32 is phosphorylated at Thr-34 mainly by protein kinase A (PKA) it becomes a potent inhibitor of protein phosphatase 1 (PP1), which in turn regulates the phosphorylation state of several classes of proteins, including transcription factors, ionotropic glutamate receptors and ion channels [66], [67]. PKA also phosphorylates and activates protein phosphatase 2A (PP2A), which dephosphorylates DARPP-32 at Thr-75 [68], [69]. Conversely, when phosphorylated at Thr-75 by Cdk5, DARPP-32 becomes an inhibitor of PKA activity, thereby relieving inhibition of PP1 [67], [70]. Therefore, regulation of the state of DARPP-32 phosphorylation provides a mechanism for integrating information at striatal medium-sized spiny neurons via a variety of neurotransmitters and it may play important regulatory functions in motor behavior [65], [71]–[74]. Given that the striatum is an important target of Mn toxicity [5], [6] and that Mn may disrupt dopaminergic and glutamatergic transmission [35], [75], we hypothesized that Mn mediates its neurotoxic effects, at least in part, via disruption of DARPP-32 regulation. Notably, changes on DARPP-32 phosphorylation by Mn have yet to be described either *in vitro* or *in vivo*.

The biological consequences of developmental Mn exposure may be particularly harmful, affecting neurogenesis, learning and memory, with predisposition to late onset neurodegenerative disorders. Exposure to a toxic stimulus may result in “imprinting”, a process by which early environmental factors may permanently alter an organism's gene expression profile [11].

Taken together, the present study was designed to assess alterations in cell signaling pathways, production of oxidative stress and later-life behavioral deficits as a consequence of a short-term postnatal Mn exposure (PN8-12). For the first time, we establish Mn-induced disruption in regulatory mechanisms of ERK1/2, AKT and DARPP-32 in the developing rat striatum. Moreover, we show an increase in oxidative stress and impairment in motor activity in immature rats exposed to this metal.

## Methods

### Ethics Statement

Wistar rats of both genders were obtained from the Universidade Federal de Santa Catarina breeding colony. The animals were maintained in an air-conditioned room (23 ± 1°C) on a 12 h light/dark cycle with water and food available ad libitum. They were treated, manipulated and euthanized according to the “Principles of Laboratory Animal Care” (NIH publication no. 80–23, revised 1996) and approved by the Committee on the Ethics of Animal Experiments of the Federal University of Santa Catarina (CEUA/UFSC; www.ceua.ufsc.br; Permit Number: PP00345). All efforts were made to minimize the number of animals used and animal suffering.

### Reagents

Primary antibodies anti-ERK1/2, anti-p38^MAPK^, anti-JNK1/2 and manganese chloride were obtained from Sigma (St. Louis, MO, USA). Anti-phospho-CREB, anti-CREB, anti-phospho-ERK1/2, anti-phospho-p38^MAPK^, anti-phospho-JNK1/2, anti-AKT, anti-phospho-AKT, anti-DARPP-32, anti-phospho-DARPP-32-Thr-34 and anti-phospho-DARPP-32-Thr-75 antibodies and LumiGlo detection reagent were purchased from Cell Signaling (Beverly, MA, USA). Anti-β-actin was purchased from Santa Cruz Biotechnology (Santa Cruz, CA, USA). Secondary antibody (anti-rabbit and anti-mouse IgG-horse radish peroxidase (HRP)-conjugated was purchased from Millipore (Temecula, CA, USA). Acrylamide, bis-acrylamide, β-mercaptoethanol, Hybond™ nitrocellulose, Amersham Hyperfilm™ ECL, sodium dodecyl sulfate (SDS) and Tris were obtained from GE Healthcare Life Sciences (Piscataway, NJ, USA). 6-hydroxy-2,5,7,8-tetramethylchroman-2-carboxylic acid (Trolox™) were obtained from Calbiochem (La Jolla, CA, USA). The N-acetyl-Asp-Glu-Val-Asp-7-amino-4-methylcoumarin (DEVD-AMC) was obtained from Biomol (Plymouth Meeting, PA, USA). 2′,7′-dichlorofluorescein diacetate (DCFH2-DA) was purchased from Invitrogen (Carlsbad, CA, USA). All other reagents were of the highest analytical grade.

### Animals and treatments

Litters were obtained from random crosses between several breeding males and females derived from the UFSC breeding colony. At birth, the number of pups was randomly culled to eight pups per litter. The treatments began when the pups were eight days old (PN8). The litters were randomly assigned to each experiment and the treatments were carried out at the same time every day (2:00 PM). The animals were individually identified for each treatment.

For the Mn exposure protocol, four to twelve groups (each group containing eight pups from a different litter) were used for the analysis. We performed four treatments (control and Mn 5, 10 and 20 mg/kg) with two animals in each of the groups. Accordingly, each experimental group was composed of eight pups treated in duplicate. The pups were treated for 5 consecutive days (8^th^ to 12^th^ postnatal day; PN8-12) with one daily intraperitoneal (i.p.) injection of saline (NaCl, 0.9%; control) or MnCl_2_ (5, 10 and 20 mg/kg, diluted in saline), as described previously [76], [77]. Rats were euthanized by decapitation on the 14^th^ postnatal day (PN14) [76], [77] and the structures of interest were dissected out for neurochemical analyses. Briefly, the brain was carefully removed, the cerebral hemispheres were divided and the hippocampi were isolated (using two fine brushes). This procedure was followed by displacement of the cortex and striatum. The striatum was carefully transected and it was separated from the nearby structures with a sharp spatula. In addition, to verify the involvement of oxidative stress in Mn-induced neurotoxicity, pups were treated with the antioxidant 6-hydroxy-2,5,7,8-tetramethylchroman-2-carboxylic acid (Trolox™) concomitantly with Mn. The same protocol as previously described was carried out with two pups in each treatment group: saline (0,9% NaCl; control), Trolox™ 1 mg/kg, MnCl_2_ 20 mg/kg, MnCl_2_ 20 mg/kg plus Trolox™ 1 mg/kg (administered 10 min before Mn). All treatments were carried out by i.p. injection. The pups' body weights were measured daily from PN8 to PN14 and the weight-gain (g) are reported as mean ± S.E.M.

### Brain metal analyses

Tissue Mn and Fe concentrations were measured by atomic absorption spectroscopy (Varian AA240™, Varian Inc., Palo Alto, CA, USA) [78]. Striatum, hippocampus and cerebral cortex were digested for 48–72 h in a sand bath (60°C) in ultrapure nitric acid (1∶10 wt/vol dilution). One hundred µl of digested tissue was brought to 1 ml of total volume with 2% nitric acid and analyzed for Mn and Fe. The mixture was then centrifuged and the supernatant was used for analysis (100 µl aliquot brought up to a 1 ml volume with 2% nitric acid). Bovine liver (10 µg Mn/l) was digested in ultrapure nitric acid and used as an internal standard. The data are expressed as µg metal/g tissue and the values are reported as mean ± S.E.M.

### Western blotting

Striatum was dissected, mechanically homogenized in 500 µl of sample buffer (200 mM Tris, 40 mM EDTA, 4% SDS, pH 6,8) and immediately boiled for 5 min. Sample dilution solution (1∶4 vol/vol; 40% glycerol, 50 mM Tris and minimal bromophenol blue) and β-mercaptoethanol were added to each sample for a final concentration of 5%. Protein content was estimated at 750 nm wavelength and the concentration calculated using a standard curve with bovine serum albumin [79]. The samples (60 µg of total protein/track) were separated by SDS-PAGE (miniVE Vertical Electrophoresis System™, GE Healthcare Life Sciences, Piscataway, NJ, USA) using 10% gels [77]. The proteins were transferred to nitrocellulose membranes using a semidry blotting apparatus (TE 70 SemiPhor™ Unit, GE Healthcare Life Sciences, Piscataway, NJ, USA) (1.2 mA/cm^2^; 1.5 h) as previously described [80]. The membranes were blocked (1 h) with 5% skim milk in TBS (10 mM Tris, 150 mM NaCl, pH 7.5). ERK1/2, JNK1/2, p38^MAPK^, AKT, CREB and DARPP-32 total and phosphorylated forms, were detected using specific antibodies incubated overnight diluted in TBS-T (10 mM Tris, 150 mM NaCl, 0,1% Tween-20, pH 7.5) containing 2.5% BSA in the dilutions 1∶1,000 (anti-phospho-CREB, anti-CREB, anti-AKT, anti-phospho-AKT-Ser-473, anti-DARPP-32, anti-phospho-DARPP-32-Thr-34, anti-phospho-DARPP-32-Thr-75, anti-phospho-JNK1/2 and anti-phospho-p38^MAPK^), 1∶2,000 (anti-phospho-ERK1/2 and anti-β-actin), 1∶10,000 (anti-JNK1/2 and anti-p38^MAPK^) or 1∶40,000 (anti-ERK1/2). Next, the membranes were incubated with anti-rabbit peroxidase-linked secondary antibody (1∶4,000) for 1 h and the reactions developed by chemiluminescence (LumiGLO®, Cell Signaling, Beverly, MA, USA). All blocking and incubation steps were followed by three washes (5 min) of the membranes with TBS-T. The optical density (O.D.) of the bands was quantified using Scion Image™ (Frederick, MD, USA). The phosphorylation level of each protein was determined as a ratio of the O.D of the phosphorylated band over the O.D. of the total band, and the data expressed as percentage of the control (considered as 100%). The values are presented as mean ± S.E.M [81].

### Histological analysis

To evaluate possible morphological changes in response to Mn treatment, rat brains were removed, fixed and processed for histological analysis by hematoxylin and eosin staining [82], [83]. Briefly, brains were removed immediately after decapitation and fixed in 4% paraformaldehyde solution for one week. After fixation, the tissues were gradually dehydrated in ethanol, cleared in xylene and embedded in paraffin. Five µm sections stained with hematoxylin and eosin. All brain areas were analyzed, and the striatum, hippocampus and cerebral cortex were imaged at ×40 magnification.

### Mitochondrial respiratory chain complexes activity

Mitochondrial fractions were prepared from the striatum [84]. The structure was homogenized in 10 volumes of 5 mM potassium phosphate buffer, pH 7.4, containing 0.3 M sucrose, 5 mM MOPS, 1 mM EGTA and 0.1% bovine serum albumin. The homogenate was centrifuged at 1,500 g for 10 min at 4°C, and the pellet was discarded. The supernatant was then centrifuged for additional 10 min at 4°C at 15,000 g to isolate the mitochondria present in the pellet, which was suspended in the same buffer. Disrupted mitochondrial fractions were obtained by freezing/thawing the samples three times. The NADH dehydrogenase (complex I), succinate dehydrogenase (complex II) and cytochrome c oxidase (complex IV) activities were analyzed [85] with the plate reader Infinite™ M200 (TECAN, Männedorf, Switzerland). The activities of the respiratory chain complexes are calculated as nmol min^−1^/mg protein^−1^ or mmol min^−1^/mg protein^−1^ ± S.E.M.

### Quantitation of ROS and F_2_-IsoPs

The production of reactive species was evaluated using the DCFH2-DA probe which is oxidized in the presence of reactive species to the DCF impermeable fluorescent compound, emitting green fluorescence after excitation at 480 nm [86]. Striata were rapidly removed and homogenized (1∶5 w/v) in a buffer containing 0.1% triton X-100, 0.12 M NaCl, 30 mM sodium phosphate, pH 7.4. Structures were homogenized using a tissue homogenizer, at 4°C followed by centrifugation at 10,000 g for 10 min. The supernatants (0.01 mg protein) were incubated with 40 nM DCFH-DA for 30 min at 37°C. The DCF fluorescence signal was measured using a Perkin-Elmer LS55 (Boston, MA, USA) spectrofluorometer using wavelengths of excitation/emission of 480/520 nm, respectively. An analytical curve was performed using a standard DCF solution to analyze the results, which were subsequently normalized as a percentage of the untreated control (100%).

Because the excessive production of ROS may induce membrane polyunsaturated fatty acid oxidation, resulting in lipid peroxidation products, such as F_2_-isoprostanes (F_2_-IsoPs), we evaluated the production of this substance in the striatum of treated animals. The F_2_-IsoPs are considered biomarkers of oxidative stress in both *in vitro* and *in vivo* models [34]. Total F_2_-IsoPs were determined with a stable isotope dilution method with detection by gas chromatography/mass spectrometry and selective ion monitoring as previously described [87], [88]. Total F_2_-IsoPs were measured in the striatum dissected from the animals exposed *in vivo* to different doses of Mn and stored at −80°C until analysis. Briefly, the striatum were homogenized in Folch solution and lipids extracted from chloroform layer by evaporation [89] and then subjected to chemical saponification using 15% KOH to hydrolyze bound F_2_-IsoPs. The homogenates were adjusted to a pH of 3, followed by the addition of 0.1 ng of 15-F_2_α-IsoP-d4 internal standard. F_2_-IsoPs were subsequently purified by C18 and silica Sep-Pak extraction and by thin layer chromatography. They were then analyzed by pentafluorobenzyl ester, a trimethylsilyl ether derivative, via gas chromatography, negative ion chemical ionization-mass spectrometry.

### Caspase activity

Caspase activity was monitored fluorometrically by the production of fluorescent AMC from DEVD-AMC fluorogenic substrate for caspase-3 and related cysteine proteases. Striatum homogenates were prepared (1∶5, w/v) in a buffer containing 10 mM HEPES pH 7.4, 42 mM KCl, 5 mM MgCl_2_, 1 mM phenylmethylsulfonylfluoride (PMSF), 0.1 mM EDTA, 0.1 mM EGTA, 1 µg/ml, pepstatin A, 1 µg/ml leupeptin, 5 µg/ml aprotinin, 0.5% 3-[(3-cholamidopropyl)-dimethyl-ammonio]-1-propanesulfonate (CHAPS), and 1 mM dithiothreitol (DTT) at 4°C. The reaction was carried out by mixing this homogenate (0.1 mg protein) to the reaction buffer containing 25 mM HEPES pH 7.4, 0.1% CHAPS, 1 mM EDTA, 10% sucrose and 3 mM DTT and was started by addition of 10 µM caspase-3 fluorogenic substrate DEVD-AMC. Cleavage of the fluorogenic substrate was detected spectrofluorometrically (Perkin Elmer LS55, Boston, MA, USA) after 2 h of incubation at 37°C, using excitation and emission wavelengths of 380 and 460 nm, respectively [90]. Fluorescence of blanks containing no fluorogenic substrate was subtracted from the values. Protein content was determined by the Lowry method [91] and caspase activity is expressed as percent of control (100%) ± S.E.M.

### Behavioral tests

The animals were kept until PN37, providing a suitable time-point for the assessment of behavioral effects of early life Mn exposure. All animals were tested in the rotarod at 22, 29 and 36 days of age (3, 4 and 5 weeks of age), and in an open field on PN37. Animals were habituated to the experiment room for 1 h prior to the initiation of the behavior tests. Behavioral tests were carried out during the light phase of the cycle (10:00–17:00 h).

### Rotarod analyses

The Rota-Rod system (Insight Equipamentos Científicos, Ribeirão Preto, Brazil) for locomotor assessment measures the time an animal maintains balance on a moving cylinder [92]. The following general conditioning and testing procedures were employed to select animals for use in the different treatment and control groups: namely, animals were first conditioned on a stationary rod for 30 seconds and during this time any animal that fell was placed back on the rotarod. Next, the animals were conditioned at a constant speed of 5 rpm for a period of 90 seconds. Animals that failed the first conditioning were allowed two additional conditioning periods, and those that failed the third conditioning period were not selected for further testing. This assured that all the animals in all the treatment groups attained an analogous a baseline (data not shown).

The same basic conditioning methodology was employed in testing treatment and control groups. Thirty minutes after the last conditioning, each animal was placed on the rotarod and its latency for falling determined. The starting speed was 5 rpm and it was increased by 0.1 revolutions per second.

### Open field analyses

To evaluate Mn-induced motor changes [92], the animals were tested in the circular open field (50 cm height ×60 cm diameter) made from acrylic (Insight Equipamentos Científicos, Ribeirão Preto, Brazil) and placed in a room with a video camera mounted on the ceiling. Each experiment lasting 10 min was recorded and the distance, average speed, number of rearing and grooming were evaluated with behavioral analysis software ANY-maze™ (Stoelting, Wood Dale, IL, USA).

### Statistical analyses

Data are expressed as mean ± S.E.M and statistical significance was assessed by one-way analysis of variance (ANOVA) followed by Duncan's or Newman-Keuls *post-hoc* test where appropriate. Kruskal-Wallis test followed by Dunn's *post-hoc* was applied to rotarod analysis. Analyses were performed with STATISTICA™ 5.1 ′98 Edition (StatSoft, Tulsa, OK, USA). A value of p ≤0.05 was considered to be significant.

## Results

### Mn exposure model

To evaluate if *in vivo* Mn treatments impaired development, we analyzed the rats' weight-gain from PN8-14. As shown in Table 1, there were no statistically significant differences between the groups, except for animals exposed to 20 mg/kg, which showed a significant decrease in weight-gain compared to controls (p<0.05; Table 1).

**Table 1:** Immature rats' weight gain exposed to Mn *in vivo*.

	Body weight PN8 (g)	Body weight PN14 (g)	Weight gain (g)
**Control**	12.68 ± 0.49	22.51 ± 0.79	9.83 ± 0.46
**5 mg/kg**	13.14 ± 0.59	23.22 ± 0.71	10.09 ± 0.41
**10 mg/kg**	13.09 ± 0.54	22.39 ± 0.66	9.30 ± 0.45
**20 mg/kg**	12.76 ± 0.42	20.96 ± 0.67	8.21 ± 0.46*

Immature rats were treated with saline (control; NaCl 0.9%) or MnCl_2_ at doses of 5, 10 or 20 mg/kg for five days (PN8-12). Body weights were measured throughout the treatment, and the weight gain recorded on PN14. Results represent mean ± S.E.M derived from eighteen independent experiments and are expressed in grams (g). Statistical analysis was performed by ANOVA followed by Duncan's test.

* p<0.05 compared to control.

**Figure 1:**
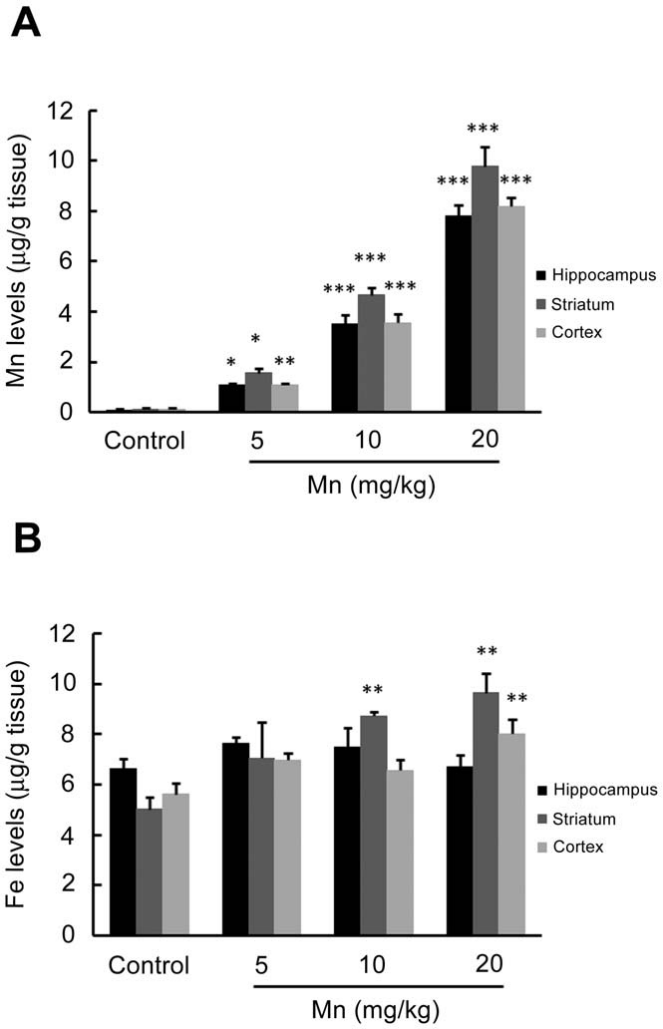
Effects of short-term Mn exposure on metal accumulation in the hippocampus, striatum and cerebral cortex of immature rats. The panels show the accumulation of Mn (**A**) and Fe (**B**). Rat pups were treated for five days (PN8-12) with saline (control; NaCl 0.9%) or MnCl_2_ at doses of 5, 10 or 20 mg/kg. The tissues were analyzed on PN14. Results represent mean ± S.E.M and are expressed in µg metal/g tissue derived from four independent experiments. Statistical analysis was performed by ANOVA followed by Duncan's test. * p<0.05, ** p<0.01, *** p<0.001 compared to control.

To characterize metal accumulation in the CNS, the striatum, hippocampus and cerebral cortex were analyzed. Mn-treated rats accumulated significantly higher Mn concentrations *vs.* controls (p<0.001) in the striatum, hippocampus and cerebral cortex (Fig. 1A). Iron (Fe) levels were also determined in the same brain regions, since disturbances in transition metals may occur secondary to Mn exposure [9]. The results showed a significant increase (p<0.01) in Fe levels only in the striatum of animals treated with 10 or 20 mg Mn/kg (Fig. 1B), and in the cerebral cortex of animals treated with 20 mg Mn/kg (Fig. 1B).

**Figure 2:**
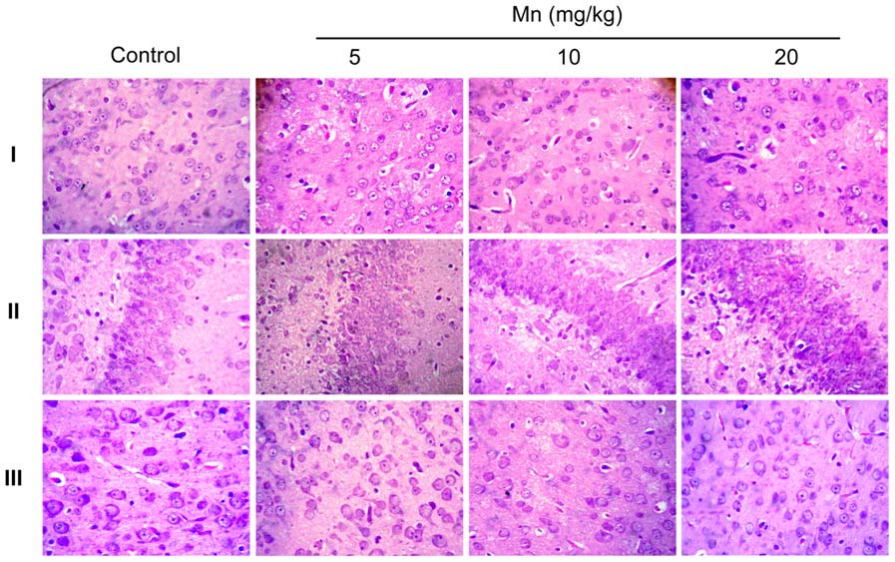
Histological evaluation of the brain of immature rats exposed to Mn *in vivo*. The panel shows representative sections from eight independent experiments of (**I**) striatum, (**II**) hippocampus and (**III**) cerebral cortex from rats treated for five days (PN8-12) with saline (control; NaCl 0.9%) or MnCl_2_ at doses of 5, 10 or 20 mg/kg. The structures analyzed on PN14. The sections were stained with hematoxylin and eosin staining. Magnification ×40.

To evaluate possible morphological changes in the brain (PN14) of rats exposed *in vivo* to Mn (PN8-12), we performed histological analysis by means of hematoxylin/eosin staining. As shown in Fig. 2, Mn treatment did not lead to overt morphological changes and cellular degeneration or death was absent. Additionally, no morphological changes were observed in the liver, kidneys, spleen or heart in Mn-treated rats (data not shown).

### Effects of Mn on cell signaling

**Figure 3:**
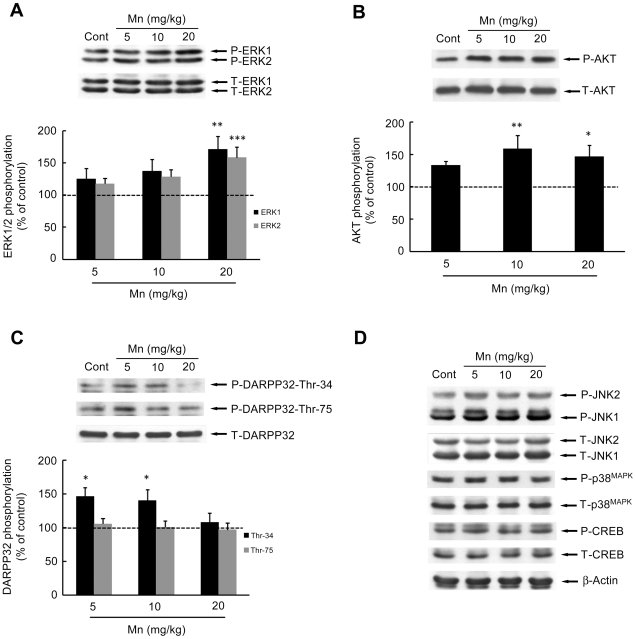
Effects of *in vivo* exposure to Mn for five days on the phosphorylation of MAPKs, AKT, CREB and DARPP-32 in the striatum of immature rats. The panels show representative immunoblotting and quantification of phosphorylation of ERK1/2 (**A**), AKT (**B**), DARPP-32-Thr-34 and -Thr-75 (**C**) and JNK1/2, p38^MAPK^, CREB and β-Actin (**D**) from rats treated for five days (PN8-12) with saline (control; NaCl 0.9%) or MnCl_2_ at doses of 5, 10 or 20 mg/kg/day. The structures analyzed on PN14. Total and phosphorylated forms of each protein were detected by specific antibodies and the reaction was developed by chemiluminescence. The phosphorylation level of each protein was determined as a ratio of the O.D. of the phosphorylated band over the O.D. of the total band and the data are expressed as percentage of the control (considered as 100%) and the values are presented as mean ± S.E.M derived from twelve independent experiments. Statistical analysis was performed by ANOVA followed by Duncan's test. * p<0.05, ** p<0.01, *** p<0.001 compared to control.

Phosphorylation and expression of MAPKs (ERK1/2, p38^MAPK^ and JNK1/2/3), AKT, CREB and DARPP-32 were evaluated on PN14 (Fig. 3). In the sub-acute model of metal exposure (PN8-12), Mn significantly increased the phosphorylation of ERK1 (71.17 ± 20.34%, p<0.01, Fig. 3A) and ERK2 (58.20 ± 16.64%, p<0.001, Fig. 3A) at the highest dose of 20 mg/kg. Additionally, the phosphorylation of AKT (Fig. 3B) significantly increased upon treatment with10 or 20 mg Mn/kg (58.79 ± 20.67%, p<0.01 and 46.61 ± 17.23%, p<0.05, respectively). DARPP-32-Thr-34 phosphorylation (Fig. 3C) was significantly increased in the lower dose Mn treated groups (46.13 ± 13.50% in 5 mg/kg, p<0.05 and 40.09 ± 15.97% in 10 mg/kg, p<0.05). However, brain DARPP-32-Thr-75 phosphorylation (Fig. 3C), as well as phosphorylation and expression of p38^MAPK^, JNK1/2/3 and CREB (Ser-133) were unchanged on PN14 (Fig. 3D).

### Oxidative stress and mitochondrial activity

**Figure 4:**
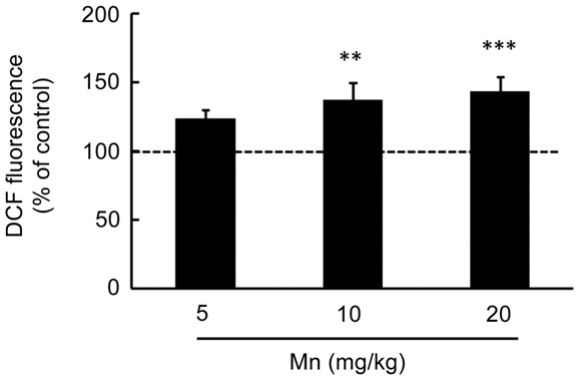
Mn induces oxidative stress in the striatum. Oxidative stress was analyzed by DCF fluorescence in the striatum of young rats treated with Mn. The graphic shows the DCF fluorescence from rats treated for five days (PN8-12) with saline (control; NaCl 0.9%) or MnCl_2_ at doses of 5, 10 or 20 mg/kg/day. The structures were analyzed on PN14. The data are expressed as percentage of the control and the values are mean ± S.E.M derived from eight independent experiments. Statistical analysis was performed by ANOVA followed by Duncan's test. ** p<0.01, *** p<0.001 compared to control.

**Figure 5:**
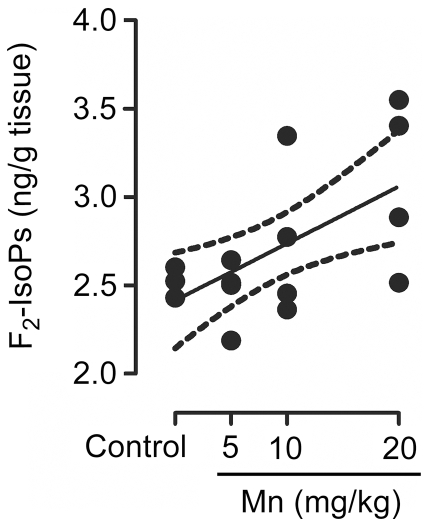
Mn induces F_2_-IsoPs production. Striatum from immature rats (PN14) exposed *in vivo* to Mn (PN8-12) were evaluated for F_2_-IsoPs levels expressed as ng/g tissue. The graphic shows a dose-dependent increase of F_2_-IsoPs levels by MnCl_2_ (5, 10 or 20 mg/kg/day). The values were obtained from four independent experiments. Pearson's correlation showed r^2^ = 0.38 with p = 0.011.

Given the involvement of oxidative stress in Mn neurotoxicity, next we measured ROS generation by DCF fluorescence and F_2_-IsoP generation. As shown in Fig. 4, Mn significantly increased ROS production in the striatum of developing rats treated with 10 or 20 mg/kg (37.21 ± 12.37%, p<0.01 and 43.07 ± 11.25%, p<0.001, respectively). Moreover, a dose-dependent increase was noted in striatal levels of F_2_-IsoPs in response to Mn treatment (Pearson's correlation r^2^ = 0.38 with p = 0.011) (Fig. 5).

**Figure 6:**
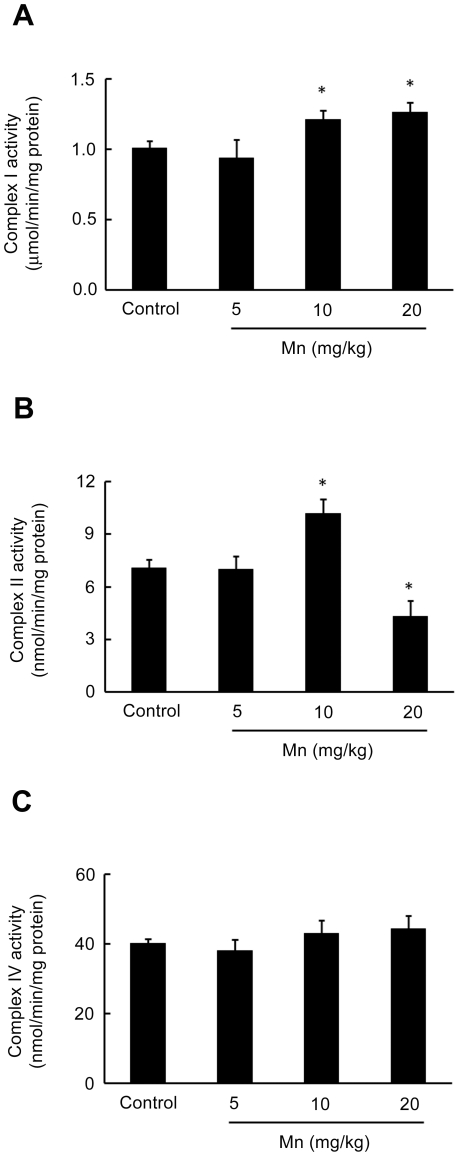
Effects of Mn on striatal activity of mitochondrial respiratory chain complexes in immature rats. (**A**) Activity of mitochondrial complex I. (**B**) Activity of mitochondrial complex II. (**C**) Activity of mitochondrial complex IV. Rat pups were treated for five days (PN8-12) with saline (control; NaCl 0.9%) or MnCl_2_ at doses of 5, 10 or 20 mg/kg. Activities were analyzed on PN14. Results represent mean ± S.E.M and are expressed as nmol min^−1^/mg protein^−1^ or mmol min^−1^/mg protein^−1^ derived from four independent experiments. Statistical analysis was performed by ANOVA followed by Duncan's test. * p<0.05 compared to control.

Mitochondrial dysfunction in response to Mn treatment was analyzed by measurements of the respiratory chain complexes I, II and IV activities. Increased complex I activity both at 10 and 20 mg Mn/kg (Fig. 6A), as well as complex II at 10 mg Mn/kg (Fig. 6B) was noted on PN14 (in pups treated with Mn on PN8-PN12). However, at 20 mg Mn/kg the complex II activity decreased (Fig. 6B). Complex IV activity was unchanged by Mn (Fig. 6C).

### Effects of Mn on caspase activity

**Figure 7:**
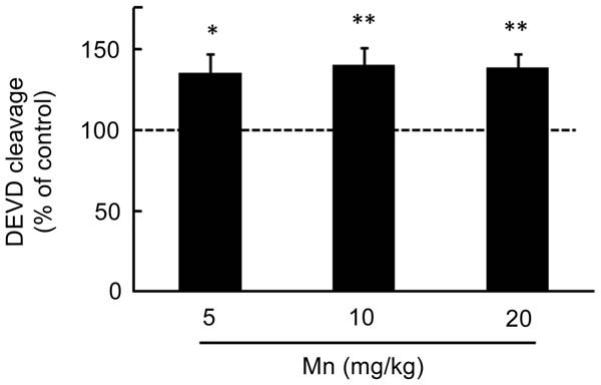
Mn treatment stimulates caspase activity in the striatum of immature rats. Caspase activities were measured by DEVD cleavage. The panel shows the DEVD cleavage test from rats treated for five days (PN8-12) with saline (control; NaCl 0.9%) or MnCl_2_ at doses of 5, 10 or 20 mg/kg/day. Activities were analyzed on PN14. Results represent mean ± S.E.M and are expressed as percentage of control (100%) derived from eight independent experiments. Statistical analysis was performed by ANOVA followed by Duncan's test. * p<0.05, ** p<0.01 compared to control.

Since, an increase in the rate of apoptosis can deregulate tissue plasticity, changing neurodevelopment and behavior later in life we decided to evaluate a possible increase in apoptosis in the striatum in response to Mn treatment, combined with analysis of neurochemical parameters. The enzymatic activity of caspase-3/7 in the striatum of immature rats exposed to Mn was evaluated by the DEVD cleavage fluorescence test. At the end of the five-day exposure period (PN14), increased caspase activity was noted with all the Mn doses (p<0.01, Fig. 7).

### Effects of Mn on motor activity

**Figure 8:**
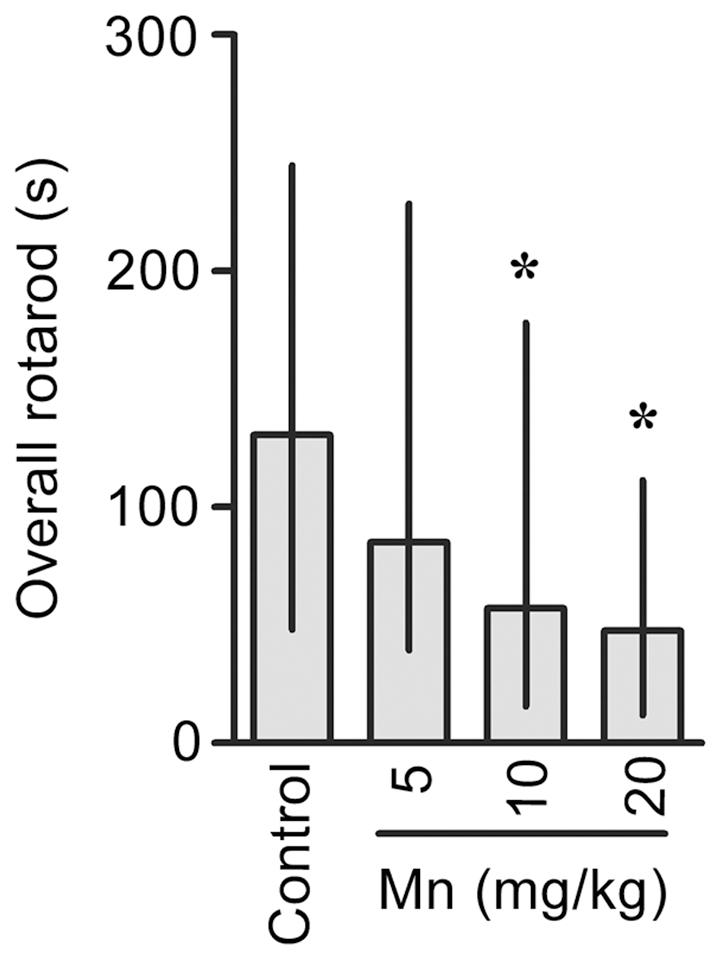
Mn exposure on PN8-12 causes later life onset motor deficits in rats. To evaluate motor coordination, control animals (saline) and rats treated with Mn (PN8-12) were tested on 22, 29 and 36 days of age (3, 4 and 5 week-old) on the rotarod task. The graphic shows the overall latency for falling in rats treated with saline (control; NaCl 0.9%) or MnCl_2_ at doses of 5, 10 or 20 mg/kg/day. Results represent median ± interquartile range and are expressed as seconds (s) to latency for falling derived from twelve independent experiments. Statistical analysis was performed by Kruskal-Wallis followed by Dunn's post-hoc test. * p<0.05 compared to control.

Mn induced a significant decrease in motor coordination in the immature rats as evaluated in the rotarod test. Control and 5 mg Mn/kg treated rats mastered the learning task, but animals treated with 10 or 20 mg Mn/kg showed decreased overall latency for falling off the rotarod *vs.* controls (p<0.05; Fig. 8).

### Effects of Trolox on Mn neurotoxicity

**Figure 9:**
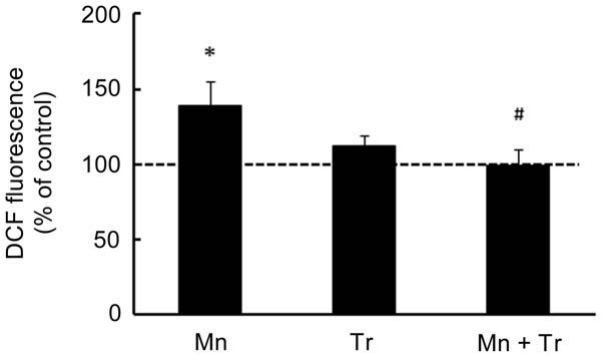
Trolox™ blocked the Mn-induced striatal oxidative stress. DCF fluorescence in the striatum from rats treated on PN8-12 with saline (control; NaCl 0.9%), MnCl_2_ 20 mg/kg (Mn), Trolox™ 1 mg/kg (Tr) or MnCl_2_ 20 mg/kg plus Trolox™ 1 mg/kg (Mn + Tr) was analyzed on PN14. Results represent mean ± S.E.M and are expressed as percent of control (100%) to DCF fluorescence derived from eight independent experiments. Statistical analysis was performed by ANOVA followed by Duncan's test. * p<0.05, compared to control; # p<0.05 compared to MnCl_2_ 20 mg/kg group.

**Figure 10:**
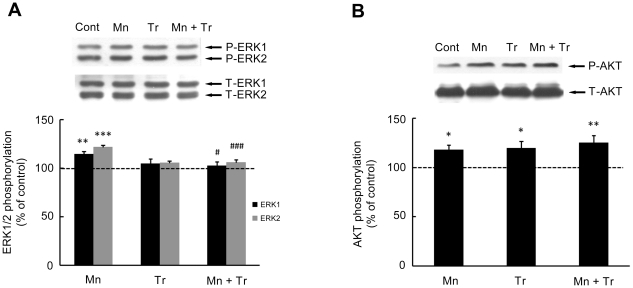
Effects of Trolox™ on the phosphorylation of ERK1/2 and AKT in the striatum of immature rats exposed to Mn. The panels (**A**) and (**B**) show representative immunoblotting and quantification of ERK1/2 and AKT phosphorylation, respectively, from rats treated for five days (PN8-12) with saline (control; NaCl 0.9%), MnCl_2_ 20 mg/kg (Mn), Trolox™ 1 mg/kg (Tr) or MnCl_2_ 20 mg/kg plus Trolox™ 1 mg/kg (Mn + Tr). The tissues were harvested from the rats on PN14. Total and phosphorylated forms of each protein were detected by specific antibodies and the reaction was developed by chemiluminescence. The phosphorylation of each protein was determined as a ratio of the O.D. of the phosphorylated band over the O.D. of the total band and the data are expressed as percentage of the control (considered as 100%) and the values are presented as mean ± S.E.M derived from twelve independent experiments. Statistical analysis was performed by ANOVA followed by Duncan's test. * p<0.05, ** p<0.01, *** p<0.001 compared to control; # p<0.05 and ### p<0.001 compared to MnCl_2_ 20 mg/kg group.

**Figure 11:**
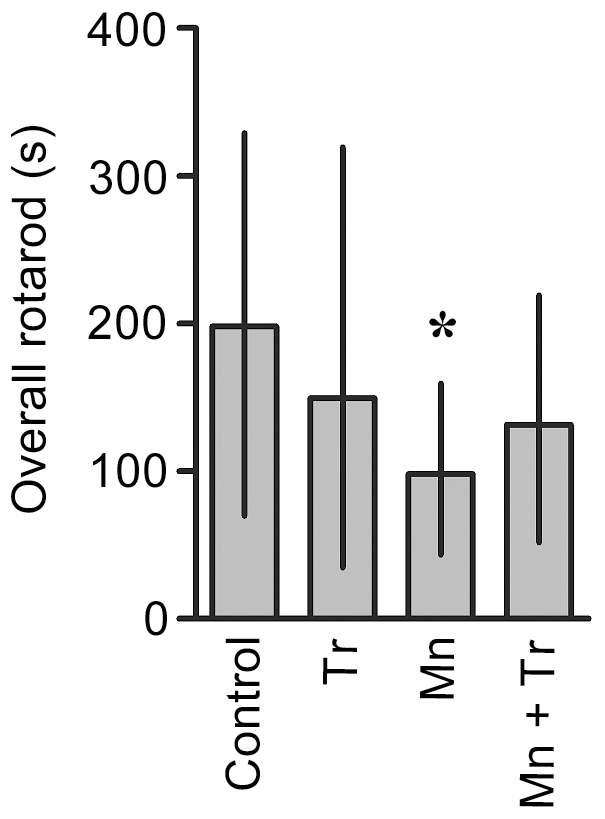
Effects of Trolox™ on the motor coordination in immature rats exposed to Mn. Rats were treated for five days (PN8-12) with saline (control; NaCl 0.9%), MnCl_2_ 20 mg/kg (Mn), Trolox™ 1 mg/kg (Tr) or MnCl_2_ 20 mg/kg plus Trolox™ 1 mg/kg (Mn + Tr), and tested on 22, 29 and 36 days of age (3, 4 and 5 week old) at the rotarod task. Results represent median ± interquartile range and are expressed as seconds (s) to latency for falling derived from twelve independent experiments. Statistical analysis was performed by Kruskal-Wallis followed by Dunn's post-hoc test. * p<0.05 compared to control.

The neurotoxic effects of Mn have been attributed at least in part to increased ROS production. Accordingly, we co-treated animals with the antioxidant Trolox™ (1 mg/kg) and Mn (20 mg/kg). As shown in Fig. 9, Trolox™ was effective in attenuating the Mn-induced increase in ROS production to levels indistinguishable from controls. Additionally, Trolox™ reversed the Mn-induced reduction in weight-gain in rats simultaneously treated with Mn (20 mg/kg) and Trolox™ (1 mg/kg), leading to weight-gain analogous to controls (Table 2). Furthermore, the Mn (20 mg/kg)-induced increase in ERK1/2 phosphorylation was blocked by Trolox™ (Fig. 10A). However, Trolox™ failed to block the Mn-induced increase in AKT phosphorylation, and unexpectedly, Trolox™ alone increased AKT phosphorylation compared to controls (Fig. 10B). Likewise, Trolox™ treatment did not mitigate the Mn-induced motor deficits (see rotarod test, Fig. 11).

**Table 2:** Protective effect of antioxidant Trolox™ on body weight gain in immature rats exposed to Mn *in vivo*.

	Body weight PN8 (g)	Body weight PN14 (g)	Weight gain (g)
**Control**	14.24 ± 0.59	24.38 ± 0.99	10.14 ± 0.66
**Mn**	13.55 ± 0.79	20.21 ± 1.01	6.66 ± 0.59***
**Tr**	13.14 ± 0.68	22.77 ± 0.87	9.63 ± 0.36
**Mn + Tr**	14.21 ± 0.86	23.20 ± 1.06	8.99 ± 0.47##

Immature rats were treated with saline (control; NaCl 0.9%), MnCl_2_ 20 mg/kg (Mn), Trolox™ 1 mg/kg (Tr) or MnCl_2_ 20 mg/kg plus Trolox™ 1 mg/kg (Mn + Tr) for five days (PN8-12). Body weights were measured throughout the treatment, and the weight gain recorded on PN14. Results represent mean ± S.E.M derived from nine independent experiments and are expressed in grams (g). Statistical analysis was performed by ANOVA followed by Duncan's test.

*** p<0.001 compared to control;

## p<0.01 compared to Mn 20 mg/kg group.

**Figure 12:**
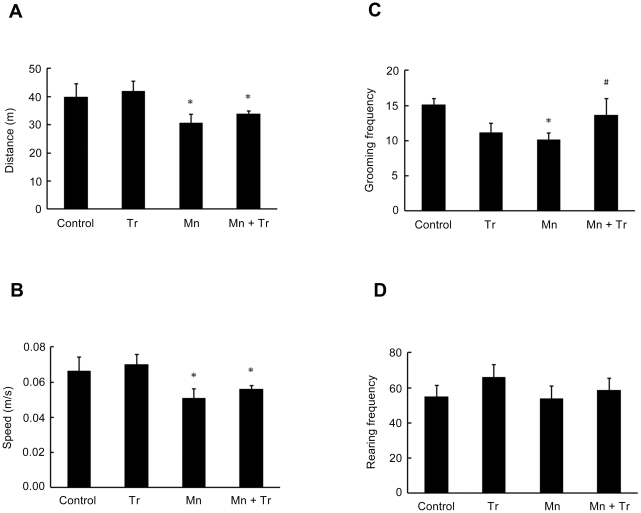
Motor behavioral effects in rats exposed to manganese and Trolox™. The animals were tested in the circular open field for 10 min to evaluate possible motor changes induced by different treatments used. The panels show the parameters analyzed from rats treated for five days (PN8-12) with saline (control; NaCl 0.9%), MnCl_2_ 20 mg/kg (Mn), Trolox™ 1 mg/kg (Tr) or MnCl_2_ 20 mg/kg plus Trolox™ 1 mg/kg (Mn + Tr). Panel (**A**) show the distance (m), (**B**) speed (m/s), (**C**) grooming frequency and (**D**) rearing frequency. Results represent mean ± S.E.M derived from twelve independent experiments. Statistical analysis was performed by ANOVA followed by Newman-Keuls test. * p<0.05 compared to control; # p<0.05 compared to MnCl_2_ 20 mg/kg group.

In the open field test, animals treated with 20 mg/kg of Mn showed a decrease in the distance traveled (p<0.05, Fig. 12A) and speed (p<0.05; Fig. 12B), as well as reduced grooming frequency (p<0.05; Fig. 12C) *vs.* controls. Treatment with Trolox™ reversed only the Mn effect on grooming frequency (p<0.05 relative to Mn, Fig. 12C). Mn treatment alone did not alter the rearing frequency (Fig. 12D).

## Discussion

Mn is an essential trace element. However, chronic or acute exposure to exceedingly high Mn levels is common and results in irreversible CNS damage [93]. Adult Mn-induced toxicity causes a neurological disorder analogous to idiopathic PD [5]. Histological observations from experimental animals and humans chronically poisoned with Mn have shown predominantly degeneration in dopaminergic nigrostriatal neurons [93]. However, a major issue, yet to be systematically addressed, relates to the neurotoxic mechanisms associated with developmental Mn exposure. While several studies have emphasized that excessive Mn concentrations in parenteral nutrition may lead to neurological disorders [6], [14], [20], [21], no *in vivo* studies have addressed the ability of Mn to interfere with intracellular signaling pathways in the developing CNS. To date, mechanisms implied in developmental Mn neurotoxicity have largely focused on oxidative stress, alterations in neurotransmitters and receptors, and behavioral abnormalities [6], [8], [9], [17], [36], [94]–[98].

The present study shows for the first time that *in vivo* neurodevelopmental sequalae of Mn exposure are associated with the modulation of intracellular signaling pathways, such as ERK1/2, AKT and DARPP-32. Moreover, oxidative stress, cell death and later-life impairment in motor function are also observed after short-term Mn exposure during a developmental period (PN8-12).

The experimental model described herein was associated with significant accumulation of brain Mn (Fig. 1A) and absent histological alterations in the brain (Fig. 2), as well as in liver, kidneys, spleen or heart (data not shown). Analyzing the numerical results of manganese levels achieved in the brain structures of immature rats, we found that baseline metal levels in the control are very low at PN14: 0.05, 0.05 and 0.06 µg Mn/g tissue in the hippocampus, striatum and cerebral cortex, respectively. In contrast, in animals treated with Mn (20 mg/kg) the levels in the same brain regions were 0.45, 0.77 and 0.33 µg/g tissue, respectively. Mn uptake into the brain is high during the neonatal developmental period that coincides with peak of brain growth. Our data agree with previous analysis performed in other studies [99]. Moreover, Mn treatment did not alter the pups' weight-gain except for the higher treatment dose (20 mg Mn/kg), where a slight decrease was noted (Table 1).

The brain structures primarily affected by Mn intoxication include the striatum, globus pallidus and substantia nigra [5], [100]. However, increased Mn levels were also noted in the hippocampus and cerebral cortex (Fig. 1A), corroborating previous studies [8], [94], [100]–[102].

A greater propensity for Mn accumulation in the developing CNS has been previously noted [5]–[7], [14]. Low Fe body-burden has been posited to represent a risk factor for Mn poisoning [98], [103], [104], with anemic states heightening sensitivity to Mn toxicity [6]. Interestingly, we observed an increase in Fe levels in the striatum of PN14 animals treated with 10 or 20 mg Mn/kg, but not in animals treated with a lower dose (5 mg Mn/kg). Moreover, in the cerebral cortex of rats treated with the highest Mn dose (20 mg/kg) we also observed an increase in Fe levels (Fig. 1B). Notably, increased Fe levels in the absence of significant changes in the levels of other metals were previously observed in the striatum, substantia nigra and cortex of juvenile mice exposed to MnCl_2_ [9]. Overall, it is possible that Mn exerts it effects on the immature brain at least in part by altering Fe metabolism. Changes in Fe concentration, may, in turn, trigger the pathophysiology of manganism. However, it is noteworthy that the lower Mn dose (5 mg/kg) produced molecular effects that were unrelated to Fe levels in the striatum, including increased Thr-34 phosphorylation of DARPP-32 and caspase activation. Notably, other studies have shown an opposite relationship, with decreased brain Fe levels in animals exposed to excessive Mn [22], [103], [105]. Moreover, increased expression of Tf in the plasma and DMT-1 transporter and TfR in the cerebellum, cortex, hippocampus, midbrain and striatum, and increased capacity of Tf bind to Fe was observed [22]. These changes may account for the increased Fe levels observed herein, reflecting a short treatment paradigm with low Mn doses. The expression or activity of the above transporters was not evaluated in the present study, and should be addressed in future studies.

Imbalances in brain metals (Mn, Fe, Cr, Cu, Zn, Co, Al) in the basal ganglia of Mn-exposed animals, behavioral changes and occurrence of oxidative stress and neuronal death have been previously noted [9], [78]. Herein, we observed increased production of ROS at 10 and 20 mg Mn/kg (Fig. 4). Moreover, F_2_-IsoPs production increased in a dose-dependent (r^2^ = 0.38) manner in the striatum (Fig. 5), consistent with reports on oxidative stress in several *in vitro* and *in vivo* models [28], [30]–[34]. ROS production may be directly related to Mn treatment or potentially is secondary to increased Fe levels and an ensuing Fenton reaction.

We observed a significant increase in mitochondrial complex I and II activities in mitochondria derived from rats exposed to 10 mg Mn/kg. Exposure to higher Mn doses (20 mg/kg) evoked distinct effects. While complex I remained increased vs. controls, complex II activity was significantly reduced by the treatment (Fig. 6). Our results differ from those reported in other studies where Mn dose-dependently inhibited respiratory chain complexes and induced ROS production in isolated rat brain mitochondria [31]. Sriram et al. [106] showed in the striatum and midbrain of rats exposed to Mn-containing welding fumes a significant reduction in mitochondrial complex proteins. We do not have a clear explanation for the apparent differences in outcomes. However, it is important to consider that mitochondrial functional capacity is age-dependent, with older animals displaying reduced mitochondrial function [107], [108]. In addition, increased mitochondrial respiration has been observed in response to stressful treatments [109] and in some neuropathological conditions accompanied by compensatory mechanisms [110]. In order to counterbalance energy deficits, mitochondrial biogenesis is stimulated via redox-sensitive transcription factors [111]. It is well established that complexes I and III are the major sites of mitochondrial reactive oxygen species generation. Thus, increased activities of complexes I and II in response to 10 mg/kg Mn treatment may lead to increased free radical production with ensuing oxidative damage to mitochondrial enzymes, including complex II, lipid membranes, as well as mtDNA [112]–[114]. Finally, the inactivation of complex II observed with the higher Mn dose might be mediated via nitric oxide (NO) derivatives such as peroxynitrite, which are formed by the controlled reaction between NO and mitochondrially-derived superoxide radical [115]. Consistent with this possibility, it has been previously shown that Mn induces NO generation [116], and that nitrosative stress inhibits complex II. Accordingly, complex II inhibition, secondary to Mn-induced mitochondrial oxidative stress, would further induce free radical production, oxidative stress and cell demise via apoptosis. In addition to the mitochondrial effects, Mn also induced an increase in striatal caspase activity (Fig. 7). Mitochondrial impairment leading to activation of apoptotic pathways is well recognized in response to Mn [30], [32], [44]. Taken together these results suggest that Mn neurotoxicity in the striatum of immature rats (PN14) likely involves both changes in the activity of mitochondrial complexes, increased ROS and F_2_-IsoPs formation, as well as the activation of caspases.

Numerous intracellular signaling pathways are modulated in response to *in vitro* Mn exposure, including: activation of ERK1/2, JNKs, p38^MAPK^ [35], [40], [42]–[44], [47], [48]. Herein, we demonstrate modification in cell signaling pathways in response to *in vivo* Mn treatment, specifically in a critical period of postnatal development. Mn exposure (PN8-12) stimulated ERK1/2 and AKT phosphorylation on PN14 (Figs. 3A and 3B), 48 h after the last Mn administration. Consistent with this temporal effect and *in vitro* and *in vivo* studies [117], [118], it is plausible that Mn treatment may lead to sustained rather than transient kinase activation. Given that signaling duration can markedly alter qualitative and quantitative features of downstream elements driving distinct cell fate decisions [119] the pattern of ERK1/2 and AKT phosphorylation may trigger long-lasting effects. The ERK and AKT pathways are involved in essential processes, such as cell proliferation, differentiation and survival [60], [120]. However, ERK1/2 may also be involved in cell death process [121], [122]. *In vitro* studies in Mn treated glial cells showed the participation of ERK1/2 and AKT in the expression of iNOS in microglia [123] and COX-2 in astrocytes [124]. In addition, it has been demonstrated that Mn produced apoptotic cell death via the ERK1/2 signaling pathway, with caspase-3 activation in PC12 and astrocytes [42], [44]. Therefore, Mn-induced ERK1/2 and AKT activation may be associated with changes in neuroplasticity and/or cell viability in the immature rat striatum, thus disturbing and impairing physiological neurodevelopment. Moreover, the oxidative stress response may be involved in the ERK1/2 activation, since the effect of Mn on ERK1/2 was abrogated by Trolox™.

The phosphoprotein DARPP-32 is highly expressed in striatal medium spiny neurons. In rodents, the level of DARPP-32 increases at birth and reaches adult levels approximately three weeks postnatally. The early and defined appearance of DARPP-32 suggests that it may influence specific aspects of neuronal differentiation and synaptogenesis [67]. Regulation of the state of DARPP-32 phosphorylation provides a mechanism for integrating information arriving at dopaminoceptive neurons in striatal medium spiny neurons via a variety of neurotransmitters. As an example, activation of D1 dopaminergic pathways stimulates DARPP-32 phosphorylation at Thr-34 (via cAMP/PKA) and thereby converts DARPP-32 into a potent inhibitor of PP1. DARPP-32 is also phosphorylated at Thr-75 by glutamatergic neurotransmission (via Cdk5 activation) and this converts DARPP-32 into an inhibitor of PKA. Therefore, DARPP-32 has the unique property of being a dual-function protein, acting either as an inhibitor of PP1 or of PKA. Alteration in the phosphorylation state of Thr-34 or Thr-75 in DARPP-32 is implicated in the generation of motor responses, as observed with psychostimulants that modulate the cAMP/PKA pathway [74]. Furthermore, in the striatum of mice PKA-dependent phosphorylation of DARPP-32 at Thr-34 by cannabinoids is related to the suppressive effects of motor activity produced by these substances [125]–[127]. In addition, in PD animal models and/or L-DOPA treatment, increased DARPP-32-Thr-34 phosphorylation and activation of ERK1/2 in the striatum are related to behavioral abnormalities [71]–[73], [125]–[127]. Despite evidence for the involvement of DARPP-32 in motor control, there are no studies on the relationship between Mn-induced motor abnormalities and modulation of DARPP-32. Accordingly, we evaluated the phosphorylation on the Thr-34 and Thr-75 sites of DARPP-32 in the striatum of developing rats (PN14) exposed to Mn (PN8-12). Interestingly, we observed increased phosphorylation of DARPP-32 at Thr-34 in PN14 rats treated with 5 or 10 mg Mn/kg (Fig. 3C). However, impairment in motor coordination activity were observed to the higher Mn doses (10 and 20 mg/kg; Fig. 8). Thus, our results suggest that the Mn-induced motor impairments likely involve additional factors to those affecting striatal DARPP-32 signaling. Regarding this possibility, some studies have shown that Mn can induce motor impairments affecting other brain areas, changing the dopamine availability and disturbing GABAergic transmission [3], [4], [102], [128]. We did not address the mechanism involved in the Mn-induced increase in Thr-34 phosphorylation in DARPP-32. However, a recent study showed PKA activation by an unknown mechanism in adult rats in response to developmental (PN1-21) Mn exposure [118]. Therefore, it is possible that a direct and/or indirect action of Mn on striatum reinforces the cAMP/PKA/Thr34-DARPP-32 pathway.

Since oxidative stress has been implied as an important factor in mediating Mn neurotoxicity, we evaluated the efficacy of antioxidant Trolox™ to mitigate the effects of Mn (20 mg/kg). The highest dose was chosen since it caused robust neurochemical and behavioral changes. Trolox™ was effective in blocking the Mn-dependent increase in ROS production (Fig. 9). Moreover, Trolox™ reversed the impairment in weight-gain observed in response to treatment with Mn 20 mg/kg (Table 2). Trolox™ co-treatment also led to a significant reduction in ERK1/2 phosphorylation (Fig. 10A), suggesting that the Mn-mediated ERK1/2 activation occurs via ROS production. However, the Mn-dependent increase in AKT phosphorylation was not altered by Trolox™ (Fig. 10B). Moreover, unexpectedly, Trolox™ itself increased AKT phosphorylation. Trolox™ also failed to reduce the Mn-dependent caspase activation (data not shown). In the behavioral test paradigms, both motor impairment (Fig. 11) and the decrease in the distance and speed in open field task (Fig. 12) inherent to Mn (20 mg/kg) treatment remained unchanged by co-treatment with Trolox™. However, Trolox™ reversed the Mn-induced reduction in grooming frequency (Fig. 12C). Taken together, we posit that increased oxidative stress and aberrant cell signaling mediate the diverse responses to Mn treatment; however, the data also suggest that several of the biochemical and behavioral alterations inherent to Mn treatment are mediated by ROS generation-independent mechanisms. Our results are consistent with previous studies where Trolox™ failed to reverse Mn-dependent hyperphosphorylation of Ser-40 on Tyrosine hydroxylase in PC12 cells, despite its ability to block H_2_O_2_ production [117].

Mn has a relatively long half-time in the CNS, consistent with a slow elimination rate [101]. In developing animals exposed to high Mn levels, Mn concentrations exceeding the homeostatic capacity may lead to an overload condition, with increased risk for neurodegenerative diseases such as Parkinson's disease at later-life stages [17]. Accordingly, the changes in vital intracellular signaling pathways, such as DARPP-32 in response to low levels of Mn treatment during a critical period of postnatal brain development, may represent important triggers of late onset neurological manifestations. Additionally, it should be considered that the DARPP-32 changes observed in response to low doses Mn treatment that were unaccompanied by immediate behavioral impairment may imprint changes in proliferation, differentiation and neural plasticity, resulting in neurological deficits at later life stages. The present study establishes changes in key intracellular signaling components which control cell survival, development and especially the complex process of neuroplasticity. Future studies should be directed at determining the relationship between early-life Mn treatment and late-onset neurological disease, and the potential for therapeutic modalities to attenuate these deficits.
